# Comparative Evaluation of Two Commercially Available Topical Gels As Adjuncts to Scaling and Root Planing for Gingival Inflammation: A Randomized Controlled Trial

**DOI:** 10.7759/cureus.116586

**Published:** 2026-09-20

**Authors:** Abhishek Sharma, Ajay Mahajan, Kanwarjit Singh Asi

**Affiliations:** 1 Periodontics, Himachal Pradesh Government Dental College and Hospital Shimla, Shimla, IND

**Keywords:** chlorhexidine, gingival index, gingivitis, metronidazole, plaque index, scaling and root planing

## Abstract

Background: Gingivitis is a common plaque-induced inflammatory periodontal disease that may progress to periodontitis if left untreated. Adjunctive topical antimicrobial agents may improve the effectiveness of conventional periodontal therapy.

Methods: This randomized controlled clinical trial included 120 participants with gingivitis or Stage I, Grade A periodontitis. Participants were randomly allocated to three groups: Group A received scaling and root planing (SRP) with placebo gel; Group B received SRP with 1% chlorhexidine gluconate (CHX) gel (CHX group); and Group C received SRP with a combination gel containing 0.5% CHX and 1.5% metronidazole (MTZ) (CHX+MTZ group). Plaque Index (PI), Gingival Index (GI), and probing pocket depth (PPD) were assessed at baseline and at 3, 6, and 12 weeks. Intergroup comparisons were performed using one-way analysis of variance and Tukey's post hoc test.

Results: All 120 participants completed the study. Baseline intergroup differences were observed for PI (p = 0.010) and GI (p < 0.001), whereas PPD was comparable (p = 0.929). At 12 weeks, both test groups had lower PI and GI scores than the placebo group. Mean PI values were 1.273 ± 0.119 in Group A, 1.229 ± 0.051 in Group B, and 1.213 ± 0.048 in Group C (p = 0.013). Mean GI values were 1.256 ± 0.097, 1.209 ± 0.053, and 1.193 ± 0.053, respectively (p = 0.003). PI and GI did not differ significantly between Groups B and C at 12 weeks. PPD also remained comparable among groups (p = 0.241).

Conclusions: Both CHX gel and the CHX-MTZ combination gel were associated with improvements in plaque control and gingival inflammation when used as adjuncts to SRP. No statistically significant difference was detected between the CHX and CHX+MTZ groups at 12 weeks. Therefore, the present findings do not establish an additional clinical advantage of adding MTZ to CHX under the conditions evaluated.

## Introduction

Periodontal diseases are inflammatory conditions initiated by bacterial biofilms that accumulate on tooth surfaces adjacent to the supporting periodontal tissues [1]. Gingivitis is the most common and earliest form of periodontal disease and affects a substantial proportion of the global population [2]. Epidemiological studies have reported the prevalence of gingivitis in dentate adults to range from 50% to 100% [3]. Gingivitis is characterized by inflammation of the gingival tissues caused by the accumulation of dental plaque and is clinically manifested by gingival redness, edema, and bleeding on probing without periodontal attachment loss [4]. The condition is often painless and may remain unnoticed by patients. Although gingivitis is reversible following effective plaque control, untreated disease may progress to periodontitis, resulting in irreversible connective tissue and alveolar bone destruction [5].

Dental plaque remains the primary etiological factor responsible for gingival and periodontal diseases [6]. The oral cavity provides an ideal environment for microbial colonization and biofilm maturation. In healthy conditions, oral biofilms are predominantly composed of Gram-positive facultative bacteria, whereas periodontal disease is associated with an increased proportion of Gram-negative anaerobic microorganisms such as *Porphyromonas gingivalis*, *Prevotella intermedia*, *Tannerella forsythia*, *Fusobacterium* species, and *Treponema* species [7]. Mechanical plaque control through toothbrushing and professional scaling remains the cornerstone of periodontal therapy [8]. However, evidence suggests that mechanical methods alone are often insufficient for achieving optimal plaque control and long-term gingival health, thereby supporting the adjunctive use of chemical antiplaque agents.

The adverse effects associated with systemic antimicrobial therapy have increased interest in topical antimicrobial agents for periodontal treatment [9]. Topical therapy offers site-specific drug delivery with reduced systemic exposure and improved patient compliance. Chlorhexidine gluconate (CHX) is considered the gold standard antiplaque agent because of its broad-spectrum antimicrobial activity and substantivity [1,10]. Metronidazole (MTZ) is another effective antimicrobial agent with activity against obligate anaerobic bacteria associated with periodontal infections [11-13]. The adjunctive use of these agents with mechanical therapy has demonstrated beneficial clinical outcomes.

Most of the previous studies have focused on antimicrobial mouth rinses and local drug delivery systems in periodontitis, while limited evidence exists regarding topical antimicrobial gels in gingivitis and early periodontal disease. Therefore, the present randomized controlled clinical trial was undertaken to evaluate and compare the effectiveness of CHX gel (1%) alone and a combination gel containing CHX (0.5%) with MTZ (1.5%) as adjuncts to scaling and root planing (SRP) in the management of gingival inflammation over 12 weeks.

## Materials and methods

Study design and ethical approval

This randomized controlled clinical trial was conducted in the Department of Periodontology, Himachal Pradesh Government Dental College and Hospital, Shimla, India. Ethical approval was obtained from the Institutional Ethics Committee (approval no. HFW(GDC)B(12)50/2015-Voll-II-2427). Written informed consent was obtained from all participants before enrollment. The trial was retrospectively registered at ClinicalTrials.gov (NCT07776197).

Study population and selection criteria

A total of 120 participants were included in the study.

Inclusion criteria included participants with age 18-50 years; gingivitis or Stage I, Grade A periodontitis; at least 20 natural teeth; a Gingival Index (GI) score ≥1 according to the Löe and Silness criteria; no active periodontal treatment during the preceding six months; and no systemic comorbidities.

Exclusion criteria included participants with smoking or smokeless tobacco use; pregnancy or lactation; orthodontic treatment; removable or fixed dental prostheses; antibiotic use within the preceding 30 days; chronic alcohol use; hematologic or immunocompromising disorders; allergy to CHX or MTZ; and use of medications associated with gingival enlargement.

Sample size calculation

The sample size was calculated using an equivalence-design formula for continuous variables with a 95% confidence level and 95% power. After allowing for 10% attrition, the required sample was 120 participants (40 per group).

Randomization and blinding

Participants fulfilling the eligibility criteria were randomly assigned into three groups using a computer-generated randomization sequence created with GraphPad Prism software (version 10.4.0; GraphPad Software, San Diego, California, USA; 2024). Group A (control group) received SRP along with placebo gel, Group B received SRP with CHX gel (1%), and Group C received SRP with a combination gel containing CHX (0.5%) and MTZ (1.5%). To ensure blinding, gel tubes were covered with opaque stickers, sealed in thick envelopes, and coded as A, B, and C by an independent individual who was not involved in treatment allocation or clinical examination. The principal investigator remained blinded to group allocation throughout the study period.

Clinical procedure and intervention

At baseline (T0), all participants underwent a complete periodontal examination that included the Plaque Index (PI), GI, and probing pocket depth (PPD). Full-mouth supragingival and subgingival SRP were completed within three days using ultrasonic and hand instruments. All clinical periodontal measurements were performed by a single examiner, and the same examiner assessed participants at baseline and at all subsequent follow-up visits. Participants were instructed to apply a pea-sized amount of the assigned gel gently to the gingiva with an index finger after brushing for approximately two minutes, twice daily for six weeks. No specific post-application interval before rinsing was prescribed in the study protocol.

Clinical parameters assessed

PI was recorded according to Silness and Löe (1964) [14], while GI was assessed using Löe and Silness (1963) criteria [15]. PPD was measured using a University of North Carolina (UNC)-15 periodontal probe at four sites per tooth (mesial, buccal, distal, and palatal/lingual), excluding third molars.

Follow-up and data collection

Clinical assessments were performed at baseline (T0), 3 weeks (T1), 6 weeks (T2), and 12 weeks (T3). Participants were instructed to avoid oral hygiene procedures and chewing gum for at least eight hours before each clinical examination. All clinical findings were recorded systematically and subjected to statistical evaluation at the end of the study period.

Statistical analysis

Statistical analyses were performed using jamovi version 2.6.26 (The jamovi project, Sydney, Australia). Continuous data are presented as means and standard deviations. Intergroup comparisons were performed using one-way analysis of variance (ANOVA), followed by Tukey's post hoc test for pairwise comparisons. Intragroup comparisons were assessed using repeated-measures ANOVA with Bonferroni correction. Categorical variables were expressed as frequencies and percentages and compared among the groups using the chi-square test. For chi-square analyses, the chi-square statistic (χ^2^), degrees of freedom (df), p-value, and Cramér's V as a measure of effect size were reported. A two-sided p-value < 0.05 was considered statistically significant.

## Results

The 120 participants were equally distributed among the three study groups (40 per group). Gender distribution did not differ significantly among groups (χ² = 0.348, df=2, p = 0.840, Cramér's V = 0.054). Mean age also did not differ significantly among groups (p = 0.095), indicating demographic comparability at baseline (Table 1).

**Table 1:** Baseline Demographic Characteristics of Study Participants ^†^ Chi-square test for gender: χ² = 0.348, df=2, p = 0.840, Cramér's V = 0.054; ^‡^ one-way analysis of variance (ANOVA). Statistical significance was set at p < 0.05. SD: standard deviation; CHX: chlorhexidine gluconate; MTZ: metronidazole

Variable	Group A (Placebo) n=40	Group B (CHX) n=40	Group C (CHX+MTZ) n=40	Total	p-value
Female, n (%)	29 (72.5)	31 (77.5)	29 (72.5)	89 (74.2)	0.840^†^
Male, n (%)	11 (27.5)	9 (22.5)	11 (27.5)	31 (25.8)	-
Age (years), mean ± SD	24.03 ± 5.99	25.20 ± 6.52	27.28 ± 7.14	-	0.095^‡^
Age range (years)	18-42	18-50	18-48	18-50	-

At baseline, statistically significant intergroup differences were observed for PI (p = 0.010) and GI (p < 0.001), whereas PPD did not differ significantly among the groups (p = 0.929) (Table 2).

**Table 2:** Intergroup Comparison of Baseline Clinical Parameters * Statistically significant at p < 0.05. PI and GI are unitless index scores; PPD is reported in millimeters (mm). SD: standard deviation; CHX: chlorhexidine gluconate; MTZ: metronidazole.

Parameter	Group A (Placebo) Mean ± SD	Group B (CHX) Mean ± SD	Group C (CHX+MTZ) Mean ± SD	p-value
Plaque Index (PI), unitless	1.579 ± 0.116	1.653 ± 0.122	1.647 ± 0.120	0.010*
Gingival Index (GI), unitless	1.546 ± 0.104	1.627 ± 0.123	1.634 ± 0.121	<0.001*
Probing pocket depth (PPD), mm	2.148 ± 0.239	2.133 ± 0.146	2.132 ± 0.148	0.929

At 12 weeks, mean PI and GI values differed significantly among the three groups (p = 0.013 and p = 0.003, respectively), with numerically highest values in Group A and lowest values in Group C. Mean PPD was also numerically lowest in Group C; however, the intergroup difference in PPD was not statistically significant (p = 0.241) (Table 3).

**Table 3:** Intergroup Comparison of Clinical Parameters at 12 Weeks * Statistically significant at p < 0.05. PI and GI are unitless index scores; PPD is reported in millimeters (mm). SD: standard deviation; CHX: chlorhexidine gluconate; MTZ: metronidazole.

Parameter	Group A (Placebo) Mean ± SD	Group B (CHX) Mean ± SD	Group C (CHX+MTZ) Mean ± SD	p-value
Plaque Index (PI), unitless	1.273 ± 0.119	1.229 ± 0.051	1.213 ± 0.048	0.013*
Gingival Index (GI), unitless	1.256 ± 0.097	1.209 ± 0.053	1.193 ± 0.053	0.003*
Probing pocket depth (PPD), mm	1.842 ± 0.351	1.780 ± 0.169	1.741 ± 0.175	0.241

## Discussion

Gingivitis is one of the most prevalent inflammatory conditions affecting the oral cavity and is primarily initiated by the accumulation of dental plaque at the gingival margin. Epidemiological studies have reported that gingivitis affects a substantial proportion of the adult population worldwide, with prevalence rates ranging from 50% to 100% [2]. Although gingivitis is considered a reversible inflammatory condition, failure to control plaque accumulation may result in progression to periodontitis, an irreversible destructive disease characterized by connective tissue attachment loss and alveolar bone destruction [16]. Therefore, prevention and early management of gingival inflammation remain essential for maintaining long-term periodontal health.

Mechanical plaque control through supragingival and subgingival SRP continues to be the cornerstone of periodontal therapy [17]. However, previous literature has demonstrated that adjunctive use of topical antimicrobial agents has been investigated for its potential to enhance plaque control and reduce gingival inflammation. The present randomized controlled clinical trial was therefore undertaken to evaluate and compare the effectiveness of CHX gel (1%) and a combination gel containing CHX (0.5%) with MTZ (1.5%) as adjuncts to SRP in the management of gingival inflammation.

In the present study, 120 participants completed the three-month follow-up period, indicating good patient compliance and acceptance of the treatment protocol. The high follow-up rate observed in this study may be attributed to increased oral health awareness, patient motivation, and accessibility to the treatment center. Previous studies have also reported that health literacy and awareness significantly influence oral health behavior, treatment compliance, and periodontal outcomes [18].

The present study demonstrated a gradual reduction in PI, GI, and PPD values in all study groups over the 12-week period. However, the reduction in PI and GI scores was more pronounced in the CHX and CHX-MTZ groups compared to the placebo group. These findings indicate greater reductions in PI and GI in the active-treatment groups than in the placebo group during the study period. The improved clinical outcomes observed in the test groups may be attributed to the antimicrobial efficacy of CHX and MTZ against periodontal pathogens [1,10,13].

It is important to note that statistically significant baseline differences were present among the study groups for PI and GI, whereas baseline PPD did not differ significantly. These baseline imbalances may have influenced the magnitude of the observed treatment effects. The statistical analysis used intergroup ANOVA and repeated-measures ANOVA, and a baseline-adjusted analysis using analysis of covariance (ANCOVA) was not performed. Therefore, intergroup comparisons should be interpreted with appropriate caution.

At 12 weeks, no statistically significant difference was detected between the CHX and CHX+MTZ groups with respect to PI and GI scores. However, the absence of a statistically significant difference should not be interpreted as evidence of equivalence between the two formulations. Within the duration and conditions of the present study, the findings did not demonstrate a statistically significant advantage of adding MTZ to CHX. Further studies specifically designed to assess equivalence or non-inferiority are required before drawing definitive conclusions regarding comparative efficacy.

CHX is widely regarded as the gold standard antiplaque agent because of its broad-spectrum antimicrobial activity and prolonged substantivity [1,10]. It is effective against both Gram-positive and Gram-negative bacteria and acts by disrupting bacterial cell membranes, thereby reducing microbial colonization and plaque formation [19]. Despite certain adverse effects associated with prolonged use, such as tooth staining and altered taste sensation, CHX remains one of the most effective topical antimicrobial agents used in periodontal therapy [11]. MTZ, on the other hand, is particularly active against obligate anaerobic bacteria and spirochetes implicated in periodontal diseases. Its cytotoxic metabolites interfere with bacterial DNA synthesis, ultimately leading to cell death [12-13].

The findings of the present study are consistent with those reported by Pradeep et al. [1], Mendigeri et al. [20], Acharya et al. [21], Mishra et al. [22], and Panhwar et al. [3], who also observed significant improvement in plaque and gingival parameters following adjunctive use of CHX and MTZ formulations in periodontal therapy. Similarly, Pedrazzoli et al. [23] and Stelzel and Flores-de-Jacoby [24] demonstrated that local MTZ therapy produced favorable clinical and microbiological outcomes in periodontal disease management. Perinetti et al. [25] further reported greater reductions in PPD with CHX and MTZ gels compared to placebo therapy.

However, a few studies have reported findings contrary to the present investigation. Lie et al. [26] observed that topical MTZ and tetracycline ointments provided clinical outcomes comparable to mechanical therapy alone. Similarly, Awartani and Zulqarnain [27] found no statistically significant difference between SRP alone and SRP combined with MTZ gel application in mild-to-moderate periodontitis. Riep et al. [28] and Kumar Singh et al. [29] also reported similar improvements between adjunctive antimicrobial therapy and mechanical debridement alone. These contradictory findings may be related to differences in study design, disease severity, antimicrobial concentration, follow-up duration, and patient compliance.

The reduction in clinical parameters observed even in the placebo group in the present study may partly be explained by the Hawthorne effect, wherein participants tend to improve oral hygiene practices and treatment compliance due to repeated examinations and increased attention during clinical trials [30]. Additionally, the beneficial effects of thorough SRP itself cannot be overlooked, as mechanical disruption and removal of subgingival biofilm remain fundamental in reducing periodontal inflammation [26-29].

Overall, adjunctive use of the topical antimicrobial gels along with SRP was associated with improvement in the evaluated clinical parameters during the study period. At 12 weeks, PI and GI differed significantly among the three groups, while the intergroup difference in PPD was not statistically significant. No statistically significant difference was detected between the CHX and CHX+MTZ groups for PI and GI. Accordingly, the present findings should be interpreted as showing no detected statistical advantage of the CHX+MTZ combination over CHX alone within the study period, rather than demonstrating equivalence between the two interventions.

Limitations

This study has several limitations. The follow-up period was limited to 12 weeks, restricting assessment of the long-term durability of the observed effects. Gel application was patient-dependent, and adherence was not objectively quantified by weighing the gel tubes before and after use. A formal quantitative assessment of intra-examiner reliability was not performed. Statistically significant baseline differences in PI and GI were present among the study groups, and a baseline-adjusted analysis using ANCOVA was not performed; therefore, these differences may have influenced the observed treatment effects. The study was conducted at a single center, which may limit generalizability, and outcomes were restricted to clinical measures without microbiological or biochemical assessment. Future multicenter studies with longer follow-up, objective adherence assessment, examiner reliability assessment, and baseline-adjusted statistical analyses are recommended.

## Conclusions

Within the limitations of this study, 1% CHX gel and the combination gel containing 0.5% CHX and 1.5% MTZ were associated with improvements in plaque control and gingival health when used as adjuncts to SRP. No statistically significant difference was detected between the CHX and CHX+MTZ groups during the 12-week study period. Therefore, the present findings do not establish an additional clinical advantage of adding MTZ to CHX under the conditions evaluated.
